# Examining associations between body appreciation and positive well-being among young adults: A cross-sectional analysis

**DOI:** 10.1177/13591053251313592

**Published:** 2025-01-30

**Authors:** Joshua A Marmara, Warwick Hosking, Siân A McLean

**Affiliations:** 1 La Trobe University, Australia; 2 Victoria University, Australia

**Keywords:** body appreciation, exercise motivations, physical activity, positive psychology, positive well-being, self-compassion

## Abstract

Interest in positive body image stems from its contrast with negative body image. Research shows self-compassion and physical activity enhance body appreciation and positive well-being, yet their interaction in young adults is not well understood. This study examined connections between self-compassion, planned physical activity and intrinsic exercise motivations in 386 adults aged 18–39 (mean age = 27.54; SD = 5.58). Path analysis and serial mediation analysis revealed positive associations between self-compassion, planned physical activity, body appreciation and positive well-being, with notable sex differences. In men, self-compassion was linked to planned physical activity through intrinsic exercise motivations, which improved positive well-being via body appreciation. For women, planned physical activity indirectly influenced positive well-being through body appreciation. These findings underscore the importance of integrating sex-specific factors into health psychology interventions aimed at promoting positive body image. They also suggest avenues for future research to enhance well-being through targeted self-compassion and physical activity strategies.

## Introduction

Historically, body image research has primarily concentrated on negative aspects, neglecting the subtleties of positive body image (Grogan, 2011). However, recent efforts have increasingly acknowledged the multifaceted nature of positive body image (Andrew et al., 2016; Linardon et al., 2022; Tylka and Wood-Barcalow, 2015a). Positive body image encompasses acceptance, love, appreciation and respect for one’s body, acknowledging its imperfections (Tylka, 2012). This shift is aligned with the principles of positive psychology and offers insights for those grappling with body dissatisfaction (Tylka, 2012). Body appreciation, defined as embracing the body’s features, functionality and health while rejecting media-driven ideals, is a well-researched aspect of positive body image (Alleva et al., 2018; Avalos et al., 2005). Building on this foundation, the present study aims to explore additional factors linked to body appreciation and positive well-being.

Extensive cross-sectional research shows positive correlations between body appreciation and aspects of positive well-being, including mood, life satisfaction, positive affect and eudaimonic well-being (Linardon et al., 2022; Webb et al., 2015). However, gaps remain in understanding other influencing factors and their effects across sexes. Self-objectification theory (Fredrickson and Roberts, 1997) offers a lens for examining these sex differences, suggesting that women, more often than men, internalise societal ideals, which may hinder body appreciation. Men, less impacted by appearance-based scrutiny, may value their bodies more for functionality (He et al., 2020; Swami et al., 2008a). This paper further examines the roles of self-compassion, intrinsic exercise motivations and planned physical activity in body appreciation and positive well-being for both men and women.

### Body appreciation and positive well-being

Recent findings suggest that men report higher levels of body appreciation than women (He et al., 2020), potentially due to more flexible appearance ideals and greater resources for fostering positive body image, such as financial means for self-care and physical activities (Swami et al., 2008a). Emerging evidence also indicates that body appreciation may offer protective benefits and serve as an intervention target for improved mental health outcomes over time (Linardon et al., 2022; Urke et al., 2021). People who appreciate their bodies may view them holistically, valuing unique characteristics while avoiding excessive focus on perceived imperfections (Avalos et al., 2005; Linardon et al., 2022; Tylka and Wood-Barcalow, 2015a), potentially disrupting the links between sociocultural pressures and body image disturbances (Stice and Bearman, 2001). Additionally, body appreciation may lead to better attunement to bodily needs and healthier behaviours (Linardon et al., 2022).

### Physical activity, body appreciation and positive well-being

One such health behaviour focused on function and capabilities rather than appearance ideals, is physical activity. Engaging in physical activity may foster body appreciation by promoting admiration of the body’s abilities, thereby enhancing positive well-being. Research has shown a direct relationship between physical activity and body appreciation (Andrew et al., 2016; Cox and McMahon, 2019; Halliwell et al., 2019), though most studies have examined activities restricted to yoga practice (containing mindfulness, akin to self-compassion; Neff, 2003) and female samples. Furthermore, a recent meta-analysis by Bassett-Gunter et al. (2017) examined the relationship between physical activity and body image in men and boys; however, none of the included studies measured body appreciation. Given that body appreciation is distinct from other constructs within the broader concept of body image (Tylka and Wood-Barcalow, 2015a), this omission highlights a lack of research focusing on body appreciation in male populations.

Research consistently demonstrates a positive correlation between physical activity and various dimensions of well-being, including increased optimism and life satisfaction across sexes (Kim et al., 2016; Maher et al., 2015). This link is attributed to factors such as enjoyment, heightened self-efficacy, improved self-concept and a greater sense of control (Berger and Motl, 2000). Notably, body appreciation may serve as a key linking factor, as regular physical activity – through purposeful movement and task execution – enhances physical capabilities like strength, endurance and flexibility, fostering a positive perception of the body (Alleva et al., 2015; Hausenblas and Fallon, 2006; Soulliard et al., 2019). This highlights the holistic benefits of physical activity for both body and mind. While yoga has been a focal point in studies promoting body appreciation and well-being, further research is needed to explore whether similar effects occur with other forms of activity, particularly those commonly undertaken by men.

### Self-compassion, physical activity and body appreciation

Self-compassion provides a theoretical framework to understand its association with body appreciation (Neff, 2003). According to self-compassion theory, individuals who adopt a self-compassionate stance experience greater self-kindness, recognise shared human experiences and maintain mindful awareness, fostering an accepting and supportive relationship with themselves (Gilbert, 2005; Neff, 2003). This mindset has been linked to higher body appreciation, as it encourages a kind and accepting response to perceived flaws and imperfections (Thøgersen-Ntoumani et al., 2017), potentially helping individuals value their physical appearance and functionality (Homan and Tylka, 2015).

Research consistently shows strong associations between self-compassion and body appreciation across sexes (Grey et al., 2022; Homan and Tylka, 2015). However, a gap remains in understanding how self-compassion and physical activity jointly influence body appreciation, particularly among young men and women. By shifting motivations for physical activity from appearance-based goals to self-care, self-compassion may foster greater focus on health and functionality, ultimately enhancing body appreciation. Given the theoretical underpinnings of self-compassion in fostering body appreciation, our model positions self-compassion as central to enhancing positive well-being (Gilbert, 1989), warranting further investigation into these dynamics.

### Intrinsic exercise motivations, self-compassion and physical activity

In addition to their independent associations with body appreciation and positive well-being, self-compassion and physical activity may contribute jointly to these outcomes. Enhanced self-compassion could encourage better self-care practices, including greater engagement in physical activity (Dunne et al., 2018; Hallion et al., 2019). This relationship likely operates through the motivations underlying physical activity, particularly intrinsic regulation (measured as intrinsic exercise motivations; Ntoumanis et al., 2021), which reflects self-determined motivation driven by personal enjoyment and satisfaction. Thus, the correlation between self-compassion and physical activity hinges on exercise motivations being fuelled by interest, enjoyment and the inherent satisfaction derived from the activity (Ntoumanis et al., 2021).

Contemporary research highlights that self-compassion promotes autonomous motivation for exercise (Ha and Ng, 2015), which is strongly associated with enhanced well-being (Wilson, 2004). This study posits that intrinsic motivation serves as a key mechanism linking self-compassion to physical activity. Self-compassion fosters kindness towards oneself and encourages authenticity and self-acceptance, which strengthen self-worth and reduce self-critical evaluation (Neff et al., 2005; Thøgersen-Ntoumani and Ntoumanis, 2006). This intrinsic motivation enables individuals to approach physical activity as a form of self-care and self-expression rather than as a pursuit driven by external validation. Notably, research among women has demonstrated a link between self-compassion and intrinsic exercise motivations (Magnus et al., 2010), yet the broader applicability of these findings across sexes remains underexplored. Examining these relationships in a multivariable framework can provide greater insight into their role in fostering positive body image and overall well-being.

### Present study

It is crucial to examine factors contributing to a positive body image in both men and women to gain a comprehensive understanding of individuals’ experiences and the construct itself (Tiggemann, 2015). This insight can inform interventions to enhance body appreciation and overall well-being. While men generally report more favourable body image perceptions, around 30% still experience body dissatisfaction (Fallon et al., 2014), with few effective interventions available (Alleva et al., 2015; Jankowski et al., 2017). Although prior research has investigated the relationships between these variables, this study uniquely examines their associative patterns within a cross-sectional framework, exploring the correlational pathways hypothesised here. To achieve this, path analysis was employed to investigate how self-compassion relates to body appreciation and positive well-being in both men and women within a single analysis, while also considering physical activity and intrinsic exercise motivations.

**Figure 1.**
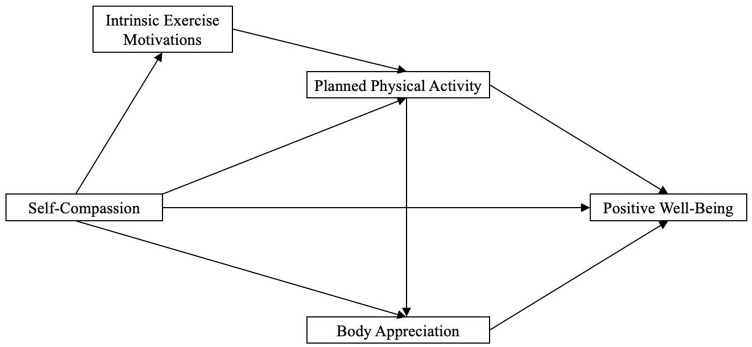
Hypothesised path model.

The aim of this study was to compare the direct and indirect relationships between self-compassion, intrinsic exercise motivations and physical activity with body appreciation and positive well-being among young men and women (see Figure 1). It was predicted that (H1) self-compassion, planned physical activity and body appreciation would be positively associated with positive well-being; (H2) self-compassion would be indirectly associated with planned physical activity through intrinsic exercise motivations; (H3) planned physical activity would be indirectly associated with positive well-being through body appreciation; (H4) self-compassion would be indirectly associated with positive well-being through body appreciation; and (H5) Self-compassion would have an indirect association with positive well-being through a sequential pathway encompassing intrinsic exercise motivations, planned physical activity and body appreciation. Lastly, the strength of model pathways between men and women was compared, with predictions (H6) that self-compassion’s pathway to body appreciation and positive well-being would be stronger for women, due to self-compassion mitigating self-objectifying tendencies; and (H7) a stronger pathway from intrinsic exercise motivations to body appreciation would be observed in men, who value physical activity for functionality rather than appearance-based reasons.

## Method

### Participants

Participants were eligible if they were between 18 and 39 years old and from English-speaking countries (Australia, Canada, Ireland, New Zealand, United Kingdom and United States of America; USA). The choice of specific countries was intentional and aimed to ensure cultural and linguistic homogeneity within the sample. This approach facilitates a more focused examination of shared experiences related to body image and well-being within the context of English-speaking societies, contributing to the coherence and generalisability of the study’s findings. A Monte Carlo power analysis was conducted to ascertain the optimal sample size required for the serial mediation analysis (Schoemann et al., 2017). Based on the continuously varying sample size, the analysis suggested that approximately 120 individuals per sex group was needed to achieve a statistical power of at least 80% for detecting the hypothesised indirect effect.

Three hundred and ninety-four participants completed an online survey after indicating informed consent. These included 216 men, 170 women and eight participants identified as non-binary. These eight participants were excluded in the present analyses targeting binary sex differences. The statistical analyses were conducted using the responses from the 386 participants who identified as either male or female (mean age = 27.54, SD = 5.58), yielding a maximum random sampling error of 0.089 for a 95% confidence interval and 0.117 for a 99% confidence interval (see Table 1). For demographic characteristics of the sample. In brief, most participants worked full-time (44.3%), had an undergraduate degree (40.4%), were from outer metropolitan suburbs (41.7%), reported Caucasian ethnicity (57.8%) and lived in the USA (54.9%).

**Table 1.** Sociodemographic characteristics of participants (*N* = 386).

Sociodemographic characteristics	Total sample	
	*n*	%
Sex		
Male	216	56.0
Female	170	44.0
Country of origin		
United States of America	212	54.9
United Kingdom	101	26.2
Canada	35	9.1
Australia	34	8.8
New Zealand	3	0.8
Ireland	1	0.3
Ethnicity		
Caucasian	223	57.8
Asian	55	14.2
European	34	8.8
Hispanic or Latino	28	7.3
African	24	6.2
Mixed	16	4.1
Indigenous or Native	4	1.0
Arabic	1	0.3
Other	1	0.3
Region		
Outer suburbs	161	41.7
Capital city/Inner suburbs	150	38.9
Rural areas	40	10.4
Regional centres	35	9.1
Highest education level		
Undergraduate university degree	156	40.4
Completed secondary school	122	31.6
Completed tertiary diploma or trade certificate	43	11.1
Postgraduate university degree	42	10.9
Some secondary school education	19	4.9
Primary school education	4	1.0
Employment		
Working full-time	171	44.3
Student	57	14.8
Working part-time	56	14.5
Unemployed	56	14.5
Working casually, sessionally or temping	24	6.2
Household duties	10	2.6
Receiving a pension/benefit	6	1.6
Other	6	1.6

### Materials

An online survey hosted by Qualtrics measured the variables of interest in young adults. Participants answered demographic questions along with measures of the variables of interest to this study as described below.

#### Body appreciation

The Body Appreciation-2 Scale (BAS-2; Tylka and Wood-Barcalow, 2015b) is an enhanced version of the original Body Appreciation Scale, with 10 items (e.g. *I respect my body*). The BAS-2 assesses how participants value their bodies and the extent to which they protect and promote a positive body image. Items are rated on a 5-point scale from 1 (*never*) to 5 (*always*). The BAS-2 was validated by Tylka and Wood-Barcalow (2015b), showing unidimensionality, good internal reliability (*α* = 0.97) and 3-week test-retest reliability (*r* = 0.90) across college and community samples. Construct validity was confirmed through correlations with body image and psychological well-being. The scale demonstrated acceptable internal reliability in the present study (*α* = 0.96 for men, *α* = 0.94 for women).

#### Self-compassion

The Self-Compassion Scale (SCS; Neff, 2003) is a 26-item self-report measure assessing self-compassion (e.g. *When things are going badly for me, I see the difficulties as part of life that everyone goes through*). The SCS includes six subscales: self-judgment, isolation, overidentification, self-kindness, mindfulness and common humanity. Items are rated on a 5-point Likert scale from 1 (*almost never*) to 5 (*almost always*), with reverse scoring for the three negative subscales. Subscale scores are averaged to compute an overall self-compassion score, where higher scores indicate greater self-compassion. The SCS has shown high internal reliability (*α* = 0.92) and test-retest reliability (*r* = 0.81; Neff, 2003). In the present study, the total scale score demonstrated strong internal reliability for both men (*α* = 0.93) and women (*α* = 0.92).

#### Physical activity (planned physical activity)

The Brunel Lifestyle Physical Activity Questionnaire (BLPAQ; Karageorghis et al., 2005; Vencato et al., 2017) measures both planned (PPA) and unplanned physical activity (UPA), with this analysis focusing on PPA (e.g. *How many times in a normal week do you engage in planned physical activity?*). PPA is assessed with six items regarding the intensity, frequency and duration of activities such as walking, cycling and soccer. Respondents rate each item on a 5-point scale from 1 (*not at all*) to 5 (*highly*). A mean score is calculated (range 1–5), with higher scores indicating greater physical activity. The BLPAQ shows good test-retest reliability (*r* = 0.90) and internal reliability (*α* = 0.90; Karageorghis et al., 2005). In the present analysis, internal reliability was high for men (*α* = 0.90) and women (*α* = 0.89).

#### Exercise motivations (intrinsic exercise motivations)

Intrinsic exercise motivations were assessed using the third iteration of the Behavioural Regulation in Exercise Questionnaire (BREQ-3; Markland and Tobin, 2004; Wilson et al., 2006). The questionnaire comprises 24 items rated on a 5-point Likert scale, ranging from 0 (*not true for me*) to 4 (*very true for me*). This analysis focused exclusively on the four items within the intrinsic regulation subscale (e.g. *I exercise because it’s fun*).

The intrinsic regulation subscale, known for its reliability (*ρ_c_* = 0.75; Cid et al., 2018) and strong internal reliability (*α* = 0.81; De Bilde et al., 2011), demonstrated acceptable internal reliability in the present study for both men (*α* = 0.83) and women (*α* = 0.82).

#### Positive well-being

The Warwick-Edinburgh Mental Well-Being Scale (WEMWBS; Tennant et al., 2007) is a 14-item tool designed to assess positive mental health. Participants rate statements like, *I’ve been feeling useful*, on a 5-point Likert scale, from 1 (*none of the time*) to 5 (*all of the time*), reflecting their experiences over the past 2 weeks. Scores range from 14 to 70, with higher scores indicating better well-being. The scale demonstrates strong internal reliability (*α* = 0.93; Marmara et al., 2018), which was consistent with the present study, where internal reliability was satisfactory for men (*α* = 0.94) and women (*α* = 0.93).

### Procedure

Approval for this research was granted by the Victoria University Human Ethics Committee (approval number: HRE19-092). Participants were recruited via Prolific.co, where eligible individuals received an email invitation to participate. The survey was anonymous and voluntary, with contact details for support services provided if needed. The average completion time was 20 minutes, and participants received £1.66 GBP ($3.00 AUD) for completing the survey.

### Analysis

Data were analysed using IBM SPSS (version 27) for descriptive and correlation analyses and RStudio (version 1.3.959) for measurement invariance (MI), path and serial mediation analyses with the Lavaan and semTools packages. Normality was assessed with skewness <3 and kurtosis <10 as acceptable thresholds (Kline, 2011). Multicollinearity was evaluated using Variance Inflation Factor (VIF) and tolerance statistics, with VIF <10 and tolerance >0.20 considered acceptable (Field, 2013). Descriptive statistics summarised the sample and measures, while correlations examined relationships among study variables.

Individual scales or subscales were treated as observed variables. The *χ*^2^; value is a conventional measure for assessing model fit, with a non-significant *p*-value (at the 0.05 level) indicating a good fit (Barrett, 2007). However, the *χ*^2^ statistic is highly sensitive to sample size, often leading to the rejection of models with larger samples (>200; Bentler and Bonett, 1980). To address this limitation, model fit was evaluated using additional indices. Models were considered to have acceptable fit if they met the following criteria: comparative fit index (CFI) ≥0.90, standardised root mean square residual (SRMR) ≤0.10 and root mean square error of approximation (RMSEA) ≤0.10 (Hu and Bentler, 1999; Swami and Barron, 2019). Good fit was defined as CFI ≥0.95, SRMR ≤0.08 and RMSEA ≤0.06 (Hu and Bentler, 1999; Swami and Barron, 2019).

Multi-group analysis using MI assessed whether the path coefficients linking self-compassion predictors to positive well-being were equivalent across sexes. Configural invariance allowed structural paths to vary freely, metric invariance constrained paths to equality and scalar invariance further constrained item intercepts to test for sex differences. Incremental differences in CFI and RMSEA values (ΔCFI>0.010, ΔRMSEA>0.015) were evaluated (Chen, 2007; Putnick and Bornstein, 2016). If full MI was not achieved, partial invariance was explored by sequentially relaxing constraints on specific parameters until model differences became non-significant (Marmara et al., 2022; Stavropoulos et al., 2018; Zarate et al., 2021).

Mediation analysis was performed using bootstrapping. According to Preacher and Hayes (2008), the bootstrapping approach is the most appropriate and reliable method in determining confidence intervals for certain indirect effects. Bootstrapping involves resampling with replacement, and the analysis employs a 95% bias-corrected confidence interval with 5000 bootstrappings in determining the significance of the indirect effects. If the confidence interval did not include zero, it was deemed statistically significant.

## Results

### Preliminary analyses

Data met normality assumptions, with skewness values ranging from −0.75 to 0.08 for men and −0.71 to 0.38 for women, and kurtosis values ranging from −0.45 to 0.35 for men and −1.00 to 0.61 for women. An examination of tolerance statistics confirmed no violations of multi-collinearity, as all values were within the acceptable range (VIF = 1.99–2.43, Tolerance = 0.56–0.77).

### Bivariate associations

Bivariate correlations were computed for men and women separately to identify associations between variables (see Table 2). Relationships among all variables across sex were positive and ranged from weak to moderate in strength. We transformed the Pearson *r* scores to Fisher’s *z* values to examine whether differences in correlations between men and women were significant (Corey et al., 1998). There were no significant sex differences among all variables except between intrinsic exercise motivations and body appreciation (men: *r* = 0.46, women: *r* = 0.31; *p* = 0.04).

**Table 2.** Correlations between the predictor, mediating and criterion variables for men (*n* = 216) and women (*n* = 170).

Variables	1	2	3	4	5
1. Self-compassion	–	0.36**	0.32**	0.57**	0.68**
2. Intrinsic exercise motivations	0.26*	–	0.61**	0.46**	0.46**
3. Planned physical activity	0.17*	0.52**	–	0.36**	0.37**
4. Body appreciation	0.67**	0.31**	0.23**	–	0.68**
5. Positive well-being	0.61**	0.34**	0.28**	0.58**	–

Coefficients above the diagonal represent correlations for the men, while those below the diagonal represent correlations among women.

** *p* < 0.01. *p < 0.05.

### Evaluation of the structural model

Before conducting the path and serial mediation analyses, we employed multi-group confirmatory factor analysis to establish configural, metric and scalar measurement invariance (MI) across sexes (see Table 3). The model in Figure 1. showed a good fit (CFI = 1.00, SRMR = 0.03, RMSEA = 0.07), enabling further multi-group analysis. The assessment of MI focused solely on the equivalence of factor loadings and intercepts to ensure that the measurement model was invariant. This step was essential to justify comparing latent constructs across men and women (Kline, 2016). The invariance of structural paths was not examined at this phase, as the primary objective was to establish MI before proceeding with path and serial mediation analyses.

**Table 3.** Tests of measurement invariance across men (*n* = 216) and women (*n* = 170).

Tests of invariance	*df*	χ^2^	*p*	CFI	ΔCFI	RMSEA	ΔRMSEA	SRMR	AIC
Configural	4	21.19	<0.01	1.00		0.07		0.03	93.19
Metric	4	21.18	<0.01	1.00	0.00	0.07	0.00	0.03	93.18
Scalar	8	14.37	0.07	1.00	0.00	0.04	0.03	0.03	86.33
Partial	7	13.69	0.06	1.00	0.00	0.04	0.00	0.03	85.69

CFI: comparative fit index; ΔCFI: incremental difference in CFI; RMSEA: root mean square error of approximation; ΔRMSEA: incremental difference in RMSEA; SRMR: standardised root mean square residual; AIC: Akaike information criterion.

The initial unconstrained model, representing configural invariance, allowed factor loadings and intercepts to vary across groups and demonstrated good fit (CFI = 1.00, RMSEA = 0.07). To test metric invariance, we constrained factor loadings to be equal across sex while allowing intercepts to vary, yielding a similarly good fit (CFI = 1.00, RMSEA = 0.07). These results indicate that the measurement model is equivalent across men and women at the metric level.

We then examined scalar invariance by constraining both factor loadings and intercepts to be equal across groups. This model fit well (CFI = 1.00, RMSEA = 0.04), though a minor incremental difference in RMSEA (greater than 0.015) between metric and scalar models suggested a small difference in model fit across sexes. To explore this non-invariance, we considered partial scalar invariance by freeing specific parameters based on modification indices (Stavropoulos et al., 2018; Zarate et al., 2021). Modification indices, which reflect the *χ*^2^ value for each parameter, identified no specific parameters requiring adjustment, indicating that the measurement model remains largely invariant across sexes (Kaplan, 2009; Kline, 2016).

These findings suggest that the model effectively captures relationships between constructs for both men and women, supporting latent construct comparability across sexes. This foundational MI validation allows for the comparative path and serial mediation analyses examining self-compassion, planned physical activity, intrinsic exercise motivations, body appreciation and positive well-being.

### Regression paths

**Figure 2.**
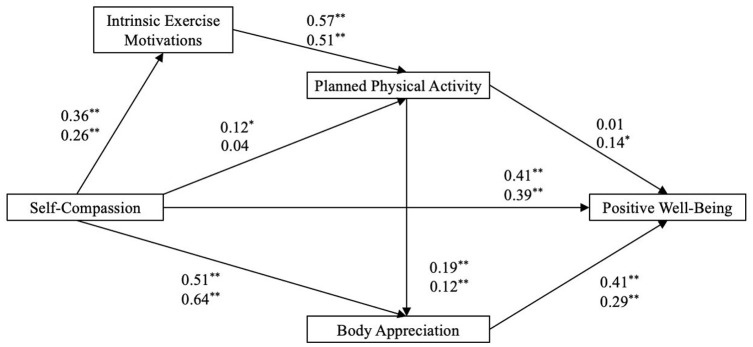
Multi-group analysis of the hypothesised path model. Standardised path coefficients are presented for men (above; *n* = 216) and women (below; *n* = 170). **p < 0.01. *p < 0.05.

For men, the model (see Figure 2) explained 41.1% of the variance in body appreciation and 41.3% in positive well-being. For women, it explained 28.7% and 39%, respectively. Self-compassion was positively and significantly associated with intrinsic exercise motivations in both sexes. However, while self-compassion had a weak but significant positive link to planned physical activity in men, no such relationship was observed in women. Notably, self-compassion showed a strong positive association with positive well-being across sexes. Additionally, intrinsic exercise motivations were significantly linked to planned physical activity for both men and women. Body appreciation was positively and significantly associated with self-compassion, planned physical activity and positive well-being in both men and women. However, the positive link between planned physical activity and positive well-being was significant only for women, with a small effect size.

### Evaluation of mediation

In the hypothesised path model (see Table 4), the indirect relationship from self-compassion to planned physical activity via intrinsic exercise motivations was significant for men but not for women. For men, the standardised estimates were small, indicating modest effect sizes. Similarly, the association between planned physical activity and positive well-being through body appreciation was significant for women (small effect size) but not for men.

**Table 4.** Mediation analyses for men (*n* = 216) and women (*n* = 170).

Sex	Pathway number	Indirect path	Unstandardised estimate	95% confidence interval (CI) [LL, UL]	*p*	Standardised estimate
Men	1.	SC → IEM → PPA	0.01	[0.01, 0.19]	0.03	0.07*
	2.	PPA → BA → PWB	4.40	[−0.64, 9.60]	0.09	0.04
	3.	SC → BA → PWB	32.54	[23.77, 41.14]	<0.01	0.17**
	4.	SC → IEM → PPA → BA → PWB	0.27	[0.08, 0.51]	0.01	0.02*
	5.	Total Model Effect	38.04	[29.59, 38.04]	<0.01	0.80**
Women	1.	SC → IEM → PPA	0.04	[−0.09, 0.15]	0.55	0.02
	2.	PPA → BA → PWB	5.14	[0.28, 10.60]	0.05	0.04*
	3.	SC → BA → PWB	23.98	[11.43, 34.59]	<0.01	0.11**
	4.	SC → IEM → PPA → BA → PWB	0.08	[−0.00, 0.21]	0.14	0.01
	5.	Total Model Effect	30.10	[14.53, 43.23]	<0.01	0.83**

SC: self-compassion; IEM: intrinsic exercise motivations; PPA: planned physical activity; BA: body appreciation; PWB: positive well-being; LL and UL indicate the lower and upper limits of the confidence intervals, respectively.

** *p* < 0.01. *p < 0.05.

Both sexes exhibited a significant indirect association between self-compassion and positive well-being via body appreciation. Among men, a sequential pathway emerged, linking self-compassion, intrinsic exercise motivations, planned physical activity, body appreciation and positive well-being. This pathway, though small, was statistically significant, suggesting that men with higher self-compassion and intrinsic exercise motivations engaged in more planned physical activity, enhancing body appreciation and well-being. For women, this pathway was weak and not statistically significant.

## Discussion

The purpose of this study was to examine the separate and combined associations of self-compassion, intrinsic exercise motivations and planned physical activity with body appreciation and positive well-being among young men and women. The first hypothesis was partially supported. While self-compassion, planned physical activity and body appreciation were positively associated with positive well-being, the specific association between planned physical activity and positive well-being was not supported for men. The second hypothesis was also partially supported as the indirect relationship from self-compassion to planned physical activity via intrinsic exercise motivations was significant for men, but not women.

The third hypothesis received partial support, as the indirect relationship between planned physical activity and positive well-being was indirectly associated with body appreciation among women, but not men. Furthermore, the relationship between self-compassion and positive well-being was indirectly associated with body appreciation among both sexes, supporting the fourth hypothesis. The fifth hypothesis received partial support as self-compassion was indirectly related to positive well-being through a sequential pathway encompassing intrinsic exercise motivations, planned physical activity and body appreciation among men, but not women. Contrary to the sixth hypothesis, men had a slightly stronger pathway from self-compassion to body appreciation and positive well-being. Last, the seventh hypothesis was supported, as men showed a stronger pathway from intrinsic exercise motivations to body appreciation, while women did not.

Examination of the model revealed several supported direct relationships. Self-compassion and planned physical activity were cross-sectionally associated body appreciation in both men and women, with higher self-compassion and greater engagement in physical activity associated with greater body appreciation. Similarly, self-compassion and planned physical activity were directly positively correlated with positive well-being, although the latter was significant only among women. Additionally, body appreciation was associated positive well-being across sexes. Consistent with previous research (Linardon et al., 2022; Tylka, 2012), our results show that higher self-compassion is linked to greater body appreciation, suggesting that self-compassion fosters a kind and accepting attitude towards oneself, extending to one’s body (Homan and Tylka, 2015). The findings also support literature indicating that engagement in planned physical activity correlates with higher body appreciation (Andrew et al., 2016; Cox and McMahon, 2019), suggesting that regular physical activity helps individuals appreciate their bodies’ capabilities and functionality. Moreover, while self-compassion and planned physical activity were directly associated with positive well-being, the latter was only significant in women. This sex-specific difference may be due to women’s motivations for physical activity, such as weight management and social interaction, which enhance self-esteem and body image, contributing to positive well-being. In contrast, men often prioritise competitive and performance-related elements, which may not impact positive well-being as directly (Jiang et al., 2021; van Uffelen et al., 2017). Last, our results indicate that body appreciation was significantly and positively associated with positive well-being, aligning with prior research emphasising its importance (Linardon et al., 2022; Tylka, 2012).

Indirect relationships within the structural model were largely supported, though sex differences were evident. Self-compassion was indirectly associated with positive well-being through body appreciation for both men and women. Planned physical activity was also indirectly associated with positive well-being through body appreciation, but only for women. Several mechanisms might explain this sex difference. Research suggests that women allocate more resources towards maintaining their physical appearance compared to men (Frederick et al., 2007; Murnen and Seabrook, 2012). This heightened investment could translate into greater psychological benefits from physical activity, as it aligns with their body image goals. Women who regularly engage in physical activity may experience increased body appreciation, thereby enhancing their overall well-being. Contrariwise, men typically invest less in their appearance, suggesting they may derive fewer psychological benefits from physical activity based on appearance-related motivations alone. Thus, while physical activity positively affects men’s well-being, the impact may be less pronounced compared to women due to lower emphasis on appearance motivations. Furthermore, self-objectification theory posits that women are more prone to internalising societal objectification, viewing themselves from an external perspective and often fixating on physical attributes. This heightened self-objectification can adversely affect women’s well-being, leading to body shame and dissatisfaction (Fredrickson and Roberts, 1997; Tiggemann and Slater, 2013). Physical activity has been shown to mitigate self-objectification among women by shifting focus towards body functionality and capabilities rather than appearance (Homan and Tylka, 2015). Women who incorporate physical activity into their lives may develop greater body appreciation, thus enhancing their overall well-being. In contrast, men are generally less susceptible to self-objectification (Rollero and De Piccoli, 2017), as they tend to focus more on functional aspects of their bodies (Calogero, 2011; Swami et al., 2008b; Tiggemann, 2015). Therefore, the impact of physical activity on reducing self-objectification and promoting a positive body image may be less significant for men compared to women. While physical activity can still contribute to men’s overall well-being through improved physical health and mood regulation, its influence on body image may be less prominent.

A sequential association was observed where self-compassion was indirectly related to positive well-being through a pathway involving intrinsic exercise motivations, planned physical activity and body appreciation among men but not women. Societal and cultural norms concerning body image and exercise could explain this pathway. Research indicates that men generally experience fewer body image concerns than women due to more flexible body-image expectations and appearance norms (Swami et al., 2008a). Moreover, men’s lower susceptibility to self-objectification allows them to value their bodies for functionality and performance, fostering intrinsic exercise motivations for reasons such as improving fitness, skill development or overall well-being (Furnham and Levitas, 2012; He et al., 2020). Intrinsic motivation is positively associated with self-compassion (Ntoumanis et al., 2021), so the significant pathway observed in men may reflect their higher levels of intrinsic exercise motivations, shaped by their lower vulnerability to appearance-related pressures. In contrast, women often face societal pressures emphasising appearance, with physical activity framed to achieve an idealised body rather than fostering well-being (Lanzarini and Cook-Cottone, 2014). As a result, women’s motivations for exercise may be more extrinsically driven, such as avoiding guilt or conforming to societal standards. These extrinsic motivations are negatively associated with self-compassion (Ntoumanis et al., 2021), weakening the pathway for women. Additionally, research has shown that men report higher levels of body appreciation than women (He et al., 2020). This higher body appreciation could contribute to a stronger feedback loop within the pathway for men. As men appreciate their bodies more, they are more likely to maintain intrinsic motives to exercise, engage in planned physical activities and consequently, experience enhanced positive well-being. Though, since body appreciation levels are generally lower for women, they may not experience the same degree of reinforcement and positive feedback via this pathway. Given the cross-sectional nature of this study, the observed associations should be interpreted with caution, as causality cannot be established. Prospective research is necessary to clarify the temporal and directional relationships within the model.

### Implications

The findings of our study offer valuable insights into the intersection of body image, self-compassion and well-being, which are critical areas of interest within health psychology. Our results suggest that interventions aimed at promoting positive body image and well-being could benefit from incorporating strategies that foster self-compassion and body appreciation, particularly in young adults. For men, health psychology interventions that emphasise self-compassion may help mitigate the negative effects of societal pressures related to body image and exercise, potentially reducing associated mental health issues. For women, promoting body appreciation through engagement in physical activities that challenge and redefine societal norms around body image could enhance psychological resilience and overall well-being, thereby reducing vulnerability to anxiety, depression and other health-related concerns. Moreover, the sex-specific differences observed in our study underscore the need for tailored health interventions that address the unique ways men and women experience and cope with body image concerns. Understanding how self-compassion interacts with body appreciation to influence positive well-being differently across sexes can inform the development of sex-sensitive interventions aimed at improving mental and physical health outcomes.

While our cross-sectional findings establish important associations, longitudinal research is essential to determine causal pathways and refine these intervention strategies. Such research could lead to more effective, evidence-based health interventions that not only improve body appreciation and self-compassion but also contribute to better mental and physical health outcomes across diverse populations. This study contributes to the body of literature in health psychology by clarifying the roles of self-compassion and body appreciation in promoting positive well-being. It highlights the importance of addressing sex-specific differences in these processes, providing a foundation for future research and intervention development that can improve health outcomes related to body image and well-being.

### Limitations and future directions

There are several limitations to this study. First, eight non-binary participants were excluded from the analysis due to the focus on binary sex differences. While our study aimed to explore sex-specific relationships between men and women, understanding these relationships among non-binary individuals is also imperative. Second, the quantitative methodology used in this project lacked depth regarding the temporal direction or reasons for associations between measured variables. A qualitative approach could further elucidate these aspects (Thornton and Lewis-Smith, 2023). Third, regarding planned physical activity, there was no objective measure of physical fitness, such as cardiorespiratory endurance, muscular strength, flexibility or agility. Thus, it remains unknown if the relationship between planned physical activity, body appreciation and positive well-being was significant due to the physical fitness of the sample. Fourth, this study did not differentiate between types of planned physical activities (e.g. football, basketball, etc.) in relation to body appreciation and positive well-being, as many participants engaged in various activities. Future research should incorporate objective measures of physical fitness and identify if specific physical activities have stronger relationships with body appreciation and positive well-being than others. Last, the cross-sectional design of this study precludes causal inferences. While the findings align with the model, this design limits the ability to establish temporal or causal relationships between the variables. Future research employing longitudinal or experimental methodologies is recommended to more robustly examine these associations and explore potential causative pathways.

### Conclusion

Our study offers significant contributions to the field of health psychology by deepening the understanding of body appreciation, self-compassion, intrinsic exercise motivations, planned physical activity and positive well-being, particularly among young men and women. This study provides novel insights by examining how intrinsic exercise motivations and body appreciation relate to psychological health and resilience, highlighting potential associative pathways between these variables. By examining sex-specific relationships within a cross-sectional framework, we identify unique associative patterns between self-compassion, intrinsic exercise motivations, planned physical activity, body appreciation and positive well-being. These insights underscore the significance of addressing sex differences and societal influences in health interventions aimed at enhancing body image and overall well-being. The findings suggest that tailored interventions, which consider the unique needs and challenges faced by young men and women, can be particularly effective in promoting psychological health. By focusing on these sex-specific factors, health psychology interventions can better support the mental and physical well-being of young adults, contributing to a more refined and effective approach to fostering resilience and positive health outcomes.
